# The impact of menopause on sexual function in women and their spouses

**DOI:** 10.4314/ahs.v20i4.56

**Published:** 2020-12

**Authors:** Zahra Bostani Khalesi, Fatemeh Jafarzadeh-Kenarsari, Yalda Donyaei Mobarrez, Mahmood Abedinzade

**Affiliations:** 1 Social Determinants of Health Research Center, Guilan University of Medical Sciences, Rasht, Iran; 2 Student Research Committee, Guilan University of Medical Sciences, Rasht, Iran; 3 Department of Physiology, School of Medicine, Guilan University Of Medical Sciences, Rasht, Iran

**Keywords:** Erectile function, female sexual function, couple, menopause

## Abstract

**Background:**

The purpose of this study is to evaluate the impact of menopause on sexual function in women and their spouses.

**Methods:**

This is a cross-sectional study that was conducted from January 2018 to May 2019 in Rasht (North of Iran). The participants included 215 menopausal women and their spouses. Data were collected using the demographic questionnaire, the Female Sexual Function Index (FSFI) questionnaire, and the International Index of Erectile Function (IIEF) questionnaire.

**Results:**

On the basis of the FSFI and IIEF scores, 36.28% (78/215) women reported female sexual dysfunction (FSD) and 17.2% (37/215) men reported erectile dysfunction (ED) with 8.37% (N = 18) being mild, 5.58% (N = 12) mild to moderate, and 3.25% (N = 7) moderate ED. After adjusting differences in the female age distribution, the total score and scores of the IIEF subscales were also not significantly lower in the spouses of women with FSD than women without FSD.

**Conclusion:**

Although, significant correlations between male erectile function and menopausal female sexual function have not identified; but, low scores of the subscales of FSFI in female participants mostly impaired sexual satisfaction and overall satisfaction in their spouses.

## Introduction

A healthy sexual life is important to humans; failure to achieve this has a negative effect on their lives^1^. Due to progress in the public health programs, average life span increased and women can, therefore, expect to live, on average, a third of their lives in post-menopausal years^2^. In Iran, nearly 3.5 million women reach menopause.^3^ By 2021, it is estimated that the number of postmenopausal women in Iran will be about 5 million 4. Menopause is an inevitable and physiological process that refers to the time when menstrual periods stop permanently^5^. Fluctuating and the deficiency of hormone levels during post-menopause can cause a change in women's sexual function 6. Approximately more than half of menopausal women suffer from low sexual desire^7^. Decreasing levels of estrogen cause vaginal dryness and dyspareunia 8. Many women face the symptoms of menopause positively and virtually pain-free; for others, the transition brings debilitating side effects, affecting the activities of daily living. Symptoms range from mild insomnia to bed-soaking night sweats, and from mild forgetfulness to depressive episodes 9. Women may be distressed due to their low desire during transient menopause because they are worried that their sex life to be treated 6. Conversely, some studies reported that the menopause brings with it a sense of sexual liberation, not having to concern themselves with an unwanted pregnancy, or worries about when they can have sex due to menstruation 10. Numerous studies have shown a decline in sexual desire and interest in menopausal women^6^, ^11^. Also, some cultures believe that women are sexually retired during menopause 12. Menopause isn't merely a harsh period for women, it's as well as difficult for their spouses. Due to the menopausal symptoms, no surprise why men are afraid of menopause 8. In a survey, 38% of spouses of menopausal women said the low sex drive due to menopause has affected their intimacy and relationship 13. It has been generally assumed that female sexual problem after menopause plays a significant role in determining a spouse's sexual function 8. Of course, Men also experience hormonal changes with aging, so that low testosterone can affect sexual desire and performance 14. Older men often have been experienced in the ED 15. ED is the incapability of a man to create an erection that's adequate for intercourse. In addition to aging, cardiovascular diseases, diabetes, education, cigarette smoking, and alcohol consumption have an association with the prevalence of ED 8. Also, attitudes and feelings toward sex, being confronted with the realities of aging, menopausal symptoms in the female partner effect on sexual life 16. Recent research has revealed that the communication behaviors of husbands and wives are interrelated 13.

Evidence to date suggests that male erectile function and female sexual function are interdependent within the context of a couple, so that FSD occurring when the women perceive ED in their partners 15. Researchers emphasized the importance to take to both members of a couple and partner into account when considering male or female sexual difficulties 2, 5, 9. In discussing the aging process on sexuality, Kaplan also noted it necessary to pay attention to each member of the couple to adapt to their partner's age-related physical and sexual changes. Since the male erectile functions and female sexual functions are interdependent, so investigate the sexual functions and dysfunctions are essential in different age groups, including postmenopausal women and their spouses 16,17. The purpose of this study is to evaluate the impact of menopause on sexual function in women and their spouses.

## Methods

This is a cross-sectional study that was conducted from January 2018 to May 2019 in Rasht (North of Iran). The present study was done to assess erectile function in men with female partners experiencing menopause. The participants included 215 menopausal women and their spouses. A multi-stage sampling technique was used to select the study participants. The inclusion criteria were as follows: Iranian nationality; Residence in Rasht; Natural menopause (as determined after 12 successive months of amenorrhea); Married and living with her spouses. The project proposal was approved by the research ethics committee of Guilan university of medical sciences fund No. IR.GUMS.REC.1396.144. People who were unwilling to participate in the study; Women with a history of hysterectomy; People with a chronic disease (hypertension, diabetes, female urge urinary incontinence, and premature ejaculation); using sex hormone supplements or phytoestrogens as a type of herbal medicines; People with psychiatric disorders or prolonged mental distress were excluded from the study.

Data were collected using the demographic questionnaire, FSFI questionnaire, and IIEF questionnaire. The demographic questionnaire was made by the researchers, containing items about variables like age, educational levels, and the occupation status of women and their spouses. The content validity of the demographic questionnaire was then assessed by expert faculty members. Female sexual function was assessed with a questionnaire developed by Rosen 18. This questionnaire had 19 questions about sexual function in the last 4 weeks. Questions included: questions 1–2 were related to sexual desire; items 3–6 to sexual arousal; items 7–10 to sexual lubrication; items 11–13 to orgasm; items 14–16 to sexual satisfaction, and items 17–19 to pain assessment. The reliability of the scale and subscales was obtained by calculating cronbach's alpha coefficient estimated ≥0. 70 that showed good reliability. Mohammadi et al. confirmed that the Persian version of the FSFI is valid and reliable. Reliability of the Persian version of the questionnaire for six subscales was calculated by internal consistency using Cronbach's alpha. The correlation between questions in all subscales was >0.61 which was acceptable. The internal consistency of the whole scale was>0.85, which indicated good reliability. Furthermore, based on the sensitivity and specificity analysis, Mohammadi et al. identified a score of 28 as a desirable cutoff for the diagnosis of women with and without sexual dysfunction 19. ED was assessed using the IIEF questionnaire. IIEF, a screening questionnaire developed by Rosen et al. 20. The IIEF comprises 15 questions that are distributed into 5 subscales: erectile function (six questions), orgasmic function (two questions), sexual desire (two questions), intercourse satisfaction (three questions), and overall satisfaction (two questions). A higher total score indicates better sexual functioning

Also, a score of zero in a subscale indicates that no sexual activity in the past four weeks. The severity of ED into five categories stratified by scores based on the IIEF-EF, no ED (EF score 26 to 30), mild ED (EF score 22–25), mild to moderate ED (EF score 17–21), moderate ED (EF score 11–16), and severe ED (EF score <11).

Pakpour et al. confirmed that the Persian version of the IIEF is valid and reliable. Reliability of the Persian version of the questionnaire for each of the five subscales was calculated by internal consistency using Cronbach's alpha. The correlation between questions in all subscales was >0.70 which were acceptable. The internal consistency of the whole scle was>0.73, which indicated good reliability 21.

Summarizing categorical variables conducted by frequency distribution. The distribution of data was checked by the Kolmogorov-Smirnov test. Student's T-test or Mann-Whitney U-test were used when to compare continuous variables. To compare normally distributed variables with adjusted by other variables was used the analysis of Covariance test.

Pearson's correlation coefficients applied to correlations between total and subscales scores the FSFI and IIEF. Also, we used partial correlations to control the effect of female age. SPSS for Windows version 16.0 (SPSS Inc. Chicago, IL., USA) was used for all the statistical analysis. The significance level was considered in 0.05.

## Results

These 215 couples had mean ages of 59.95 ± 3 years (ranged 46–64) and 56.75 ± 1.7 years (ranged 51–66) for the male and female participants, respectively.

However, the two groups were not significantly different in employment status and male mean age. There were significant difference female age group and education status between the two groups of participants. The women with FSD were older.

Demographic data regarding the study's participants (215 couples) are outlined in Table 1.

**Table 1:** Demographic characteristics of the participants according to the FSD* status

Variables			FSD couples (N = 78) %	Non-FSD couples (N =137) %	*P* value
**Mean age (y),** **Mean (SD)**	Men		60.2 ± 3.4	59.7 ± 2.6	0.061
	Women		59.1 ± 1.2	54.4 ± 2.2	<0.01
**Age** **group(Women)**	45–49 years		5(6.42)	8(5.84)	<0.01
	50–54		21(26.9)	66(48.17)	
	55–59		45(57.7)	60(43.8)	
	>60		7(8.98)	3(2.19)	
**Education Status**	Men	Primary or Secondary School	12(15.38)	25(18/25)	0.04
		High School	23(29.49)	39(28/46)	
		Diploma	38(48.71)	57(41/6)	
		University education	5(6.42)	16(11/67)	
	Women	Primary or Secondary School	9(11/54)	29(21/16)	0.02
		High School	34(43/58)	32(23/36)	
		Diploma	27(34/62)	55(40/15)	
		University education	8(10/26)	21(15/33)	
**Employment** **Status**	Men	Unemployed	4(5/13)	3(2.19)	0.08
		Government Employee	23(29/49)	41(29.93)	
		Self Employed	51(3/72)	93(67.88)	
	Women	Housewife	41(52/56)	87(63.5)	0.06
		Government Employee	9(11.54)	14(10.21)	
		Self Employed	28(35/9)	36(26.27)	

FSD = Female Sexual Dysfunction

FSD defined by FSFI score < 28

According to the total FSFI scores, 36.28% (78/215) of the women had female sexual dysfunction. On the basis of the IIEF-EF scores, 17.2% (37/215) of the men had ED with 8.37% (N = 18) being mild, 5.58% (N = 12) mild to moderate, and 3.25% (N = 7) moderate ED. Table 2 shows that there are no correlations between the FSFI and IIEF total scores among the 215 sexually active couples. Although there is a high correlation between intercourse satisfaction and overall satisfaction of IIEF scores and FSFI subscales scores (Table 2).

**Table 2:** The correlation between the FSFI and IIEF subscales

FSFI Subscales								
		Desire	Arousal	Lubrication	Orgasm	Satisfaction	Pain	Total scores
**IIEF Subscales**	**Erectile Function**	0.02	0.05	0.04	0.07	0.06	0.05	0.09
	**Intercourse Satisfaction**	0.28***	0.07	0.12*	0.17*	0.26**	0.13*	0.21*
	**Sexual Desire**	0.04	0.03	0.04	0.06	0.09	0.02	0.02
	**Orgasmic Function**	0.07	0.02	0.09	0.07	0.02	0.08	0.03
	**Overall Satisfaction**	0.25**	0.13*	0.27**	0.11*	0.28**	0.21**	0.29**
	**Total scores**	0.06	0.09	0.08	0.05	0.08	0.02	0.06

* P < 0.05

** P < 0.01

*** P < 0.001

In a female age-adjusted model, EF in partner's women without FSD is better than the partner's women with FSD, although not statistically significant (Table 3).

**Table 3:** Comparison of IIEF scores between spouses of female participants with and without FSD

IIEF Subscales	Spouses of women with FSD (N =78) (Mean ±SD)	Spouses of women without FSD (N =137) (Mean ±SD)	P-value*
**Erectile Function**	13.8 ± 5.3	15.5 ± 7.8	0.21
**Intercourse Satisfaction**	3.7 ± 1.8	4.9 ± 4.7	0.11
**Sexual Desire**	4.9 ± 2.6	5.3 ± 1.8	0.23
**Orgasmic Function**	5.8 ± 3.5	6.7 ± 3.3	0.28
**Overall Satisfaction**	5.6 ± 2.4	6.1 ± 2.8	0.17
**Total scores**	33.8 ± 3.1	38.5 ± 2.3	0.12

ED: Erectile Dysfunction defined according to IIEF total scores < 26

FSD: defined according to FSFI total scores <28 and < 3.6 in per subscales

## Discussion

The results of the present study showed that 36.28% of the female participants are suffering from sexual dysfunction. Several studies have shown that women after menopause avoid sexual relationships to reduce vaginal burning during sexual intercourse 11, 22.

Consequences of the menopausal estrogen decline include urogenital atrophy, vaginal dryness, and decreased tissue elasticity all of which can result in dyspareunia and affect sexual behavior in women 23. The difference between the obtained results about the prevalence of FSD in our study and other studies can be due to different age groups and differences in racial and cultural aspects, sample size, attitudes of these women to the menopause phenomenon and the study inclusion criteria.

The present study observed that intercourse and overall satisfaction of IIEF subscales in men were related significantly to the aspects of pain, desire, arousal, lubrication, orgasm and sexual satisfaction of their female partners. A cross-sectional study of 632 couples in Taiwan has shown correlations between mild to moderate (EF scores 17 to 21) in male participants and their partners' scores on FSFI 24. Based on these results, it can be hypothesized that having a partner who suffers from FSD leads to decreased satisfaction and a subsequent loss of intimacy in the relationship, in part due to decreased sexual activity and thereby loss of important intimacy. Several surveys asked men how to wives' menopausal symptoms affected their sexual lives. The men commonly say wives' menopausal symptoms affect their overall marital relationship, particularly if they have not a deeper level of real intimacy 25,26,27. There is a decline in sexual activity of men after a decline in sexual desire, arousal, and orgasm function and decreased sexual satisfaction in their partners 28. ED and early ejaculation to be associated with distressing orgasm problems and delayed ejaculation to be associated with distressing lubrication problems 29.

The erectile function (quality of erection, efficacy, consistency of responses, speed of onset of the erection, duration of action) of male partners of women with FSD quite different from those women without FSD^14^,^30^. According to the results of studies by researchers, the decrease in men's sexual function and satisfaction in response to the partner's FSD might be due to the man withdrawing or the attitude towards menopause 14,24, 31. A positive attitude by men about the changes experienced by menopausal women fosters the increase of emotional support for their wives, which improves the quality of sexual relations 32.

Partner factors that impact on ED include age, the menopausal status of female partners, partner interest levels of sexual activity, and the period of abstinence from sex 14, 24. Furthermore, several factors have an impact on ED such as age, the desired frequency of sexual relations, the duration of ED, and the dynamics of his sexual relationship 14. Öberg and Sjögren Fugl-Meyer (2005) found a relationship between distressing sexual dysfunctions and partner's sexual dysfunctions, especially erectile dysfunctions. They also reported an association between satisfaction with partner relationship and sexual interest, lubrication, and orgasm 33. In addition, the severity of the men's ED was associated with the frequency of orgasm and sexual satisfaction in their female partners 34.

## Conclusion

Conclusions from this study suggest that FSD not perform a major contributing factor to ED; but, female participants who had a low score of the FSFI subscales to be mostly impaired intercourse satisfaction and overall satisfaction in their spouses. These results contribute further document to the framework of literature about the interdependence of female and male sexual contacts and supported the importance of attention to the partners of menopausal women when treating the sexual problems of menopausal women. The present study had some limitations. It was a cross-sectional study and does not allow us to make causal inferences about associations between the variables. The generalizability of the results of this study should be assumed with caution because the study subjects came from couples who had beenexual contact during the past four weeks. Therefore, it is not possible to generalize the findings to those couples who avoid sexual activity altogether.
